# Outcomes of the implementation of the computer-assisted HBView system for the prevention of hepatitis B virus reactivation in chemotherapy patients: a retrospective analysis

**DOI:** 10.1186/s40780-015-0030-7

**Published:** 2015-11-04

**Authors:** Akimasa Sanagawa, Junko Kuroda, Arufumi Shiota, Noriko Kito, Masashi Takemoto, Yoshihiro Kawade, Tetsuo Esaki, Kazunori Kimura

**Affiliations:** Department of Pharmacy, Nagoya City University Hospital, 1, Kawasumi, Mizuho-cho, Mizuho-ku, Nagoya 467-8601 Japan; Department of Hospital Pharmacy, Graduate School of Pharmaceutical Sciences, Nagoya City University, 3-1, Tanabe-dori, Mizuho-ku, Nagoya 467-8603 Japan; Department of Clinical Pharmacy, Graduate School of Medical Sciences, Nagoya City University, 1, Kawasumi, Mizuho-cho, Mizuho-ku, Nagoya 467-8601 Japan

**Keywords:** HBV reactivation, Cancer chemotherapy, Computer-assist system, Pharmacist intervention

## Abstract

**Background:**

Screening for hepatitis B virus (HBV) infection is recommended worldwide for patients receiving systemic chemotherapy in accordance with clinical guidelines, but compliance varies by country and facility. Alert systems may be useful for promoting screening, but it is unclear how effective such systems are. In this study, we investigated HBV screening procedures and their incorporation into treatment regimens following the implementation of an alert system.

**Methods:**

An alert system was introduced at our hospital in April 2012. The rates of HBV screening in the periods before and after the introduction of the alert system (September 2010 to March 2012 and April 2012 to October 2013, respectively) were investigated. We collected data on hepatitis B surface antigen (HBsAg), hepatitis B surface antibody (HBsAb), hepatitis B core antibody (HBcAb), and HBV-DNA testing in patients. As a result of this analysis, we developed a system in which pharmacists would intervene to check and confirm whether HBV screening had occurred in patients scheduled to begin treatment with chemotherapy. We named our project the “HBView” project, and the rate of HBV screening and the number of times pharmacists intervened was studied during specific time periods before and after the HBView project commenced (July 2013 to December 2013 and January 2014 to June 2014, respectively).

**Results:**

After introducing the alert system, the percentage of patients tested for HBsAb/HBcAb and HBV-DNA increased significantly, from 71.6 % to 84.9 % and from 44.5 % to 69.7 %, respectively. However, the rate of compliance with HBV testing guidelines was not 100 % after interventions. The numbers of patients who were not screened but should have been before and after the introduction of HBView were 6 and 17, respectively. Two patients at risk of HBV reactivation were identified after intervention by pharmacists; their intervention thus prevented HBV reactivation.

**Conclusions:**

Compliance with clinical HBV screening guidelines was not sufficiently improved after the introduction of the automatic alert system; however, the HBView project proved useful in reinforcing the automatic alert system.

## Background

In recent years, many cytotoxic and molecular-targeted anti-cancer drugs have been developed, increasing the complexity of chemotherapy indications and regimens. These antineoplastic agents have strong pharmacological activity, and fatal adverse events are possible if medical personnel do not follow prescription guidelines carefully. Hepatitis B virus (HBV) reactivation is thought to be one of the harmful adverse events that can arise during cancer chemotherapy. When HBV reactivation occurs, treatment of the underlying disease becomes difficult and can sometimes be fatal. Treatment with a combination of rituximab and corticosteroids, for example, is one of the risk factors for HBV reactivation. Hence, it is necessary to take precautions to avoid HBV reactivation in patients receiving chemotherapy [1].

The onset rate of fulminant hepatitis in HBV-reactivated patients is higher than that of acute hepatitis, according to a research study in Japan. Additionally, a study reported that the death rate in patients with fulminant hepatitis due to HBV reactivation was 100 % [2].

The “Guideline for the Prevention of Immunosuppressive Therapy or Chemotherapy-induced Reactivation of Hepatitis B Virus Infection” was released in Japan in 2009 [3, 4]. The contents of this guideline have since been incorporated into the Japan Society of Hepatology (JHS)’s “JSH Guidelines for the Management of Hepatitis B Virus Infection” [5]. The guidelines recommend screening all patients receiving immunosuppression and systemic chemotherapy for HBV. This recommendation is also included in guidelines recently introduced overseas, such as those by the American Association for the Study of Liver Disease [6] and the European Association for the Study of the Liver [7].

However, the importance of HBV screening is not widely understood in many countries, including Japan. Various measures have been adopted to solve this problem [8–10]. In recent years, HBV reactivation rates using computer-assist systems have been reported, and awareness of the issue has improved the rate of screening guideline enforcement [11–13]. Evaluating the sequelae of HBV reactivation is confounded by the sheer number of clinical departments, the use of multiple immunosuppressant and antineoplastic agents, and the diversity of the patients’ conditions. Alert systems are considered an effective measure to counter this problem. An alert system for HBV reactivation was introduced at our hospital in April 2012, according to which an alert message that requests an HBV inspection is displayed upon entry of a prescription for a chemotherapy regimen, followed by the automatic appearance of the inspection ordering screen. However, we hypothesized that lapses in HBV testing occur despite this alert system.

Hence, we launched a study to determine the rate of compliance with HBV testing recommendations. We named this intervention the “HBView project”, and we herein report the outcome of introducing this alert system at our hospital.

## Methods

### Alert system for HBV reactivation associated with cancer chemotherapy

**Fig. 1:**
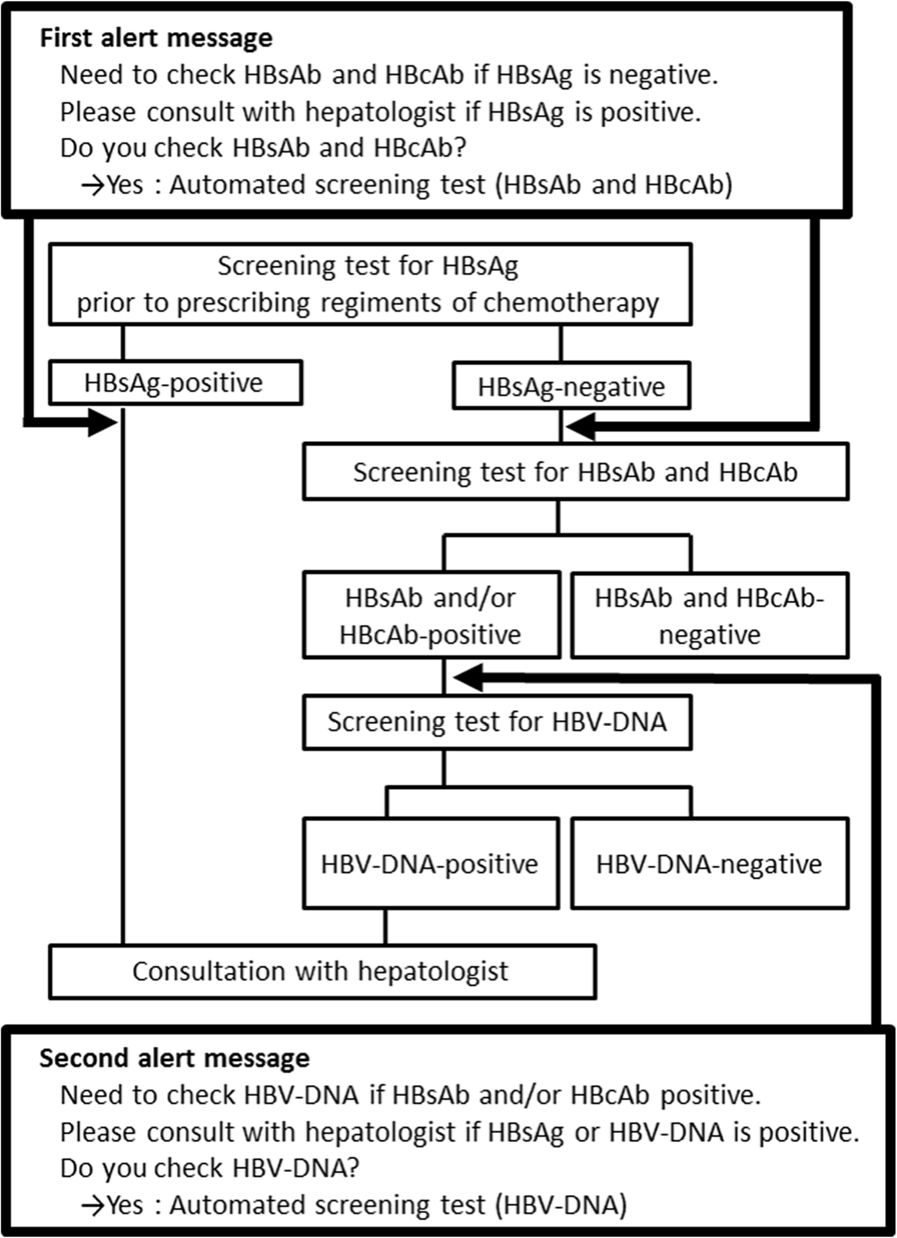
Flowchart illustrating the hepatitis B virus (HBV) screening alert system. HBsAb, hepatitis B surface antibody; HBsAg, hepatitis B surface antigen; HBcAb, hepatitis B core antibody

An alert system for HBV testing that is linked to computerized chemotherapy regimen ordering was introduced in April 2012. The displayed message recommends inspections according to the JSH Guidelines for the Management of Hepatitis B Virus Infection [5]. The computer alert message displayed when a new regimen is ordered is shown in Fig. 1. When "Yes" is chosen under the first alert message, a screen from which doctors can order the laboratory test for hepatitis B surface antibody (HBsAb) and hepatitis B core antibody (HBcAb) (using the chemiluminescence enzyme immunoassay method) appears automatically. When a second cycle regimen is ordered, a second alert message (Fig. 1) is displayed. When "Yes" is chosen, the screen from which doctors can order a laboratory test for HBV-DNA (using the real-time quantitative PCR method) appears automatically. Additionally, the “Guideline for the Prevention of Immunosuppressive Therapy or Chemotherapy-induced Reactivation of Hepatitis B Virus Infection” was available for physicians to view on the electronic medical record at the time that this alert system was introduced. The information in the alert system is revised in accordance with any revisions in the guideline.

### HBView project

**Fig. 2:**
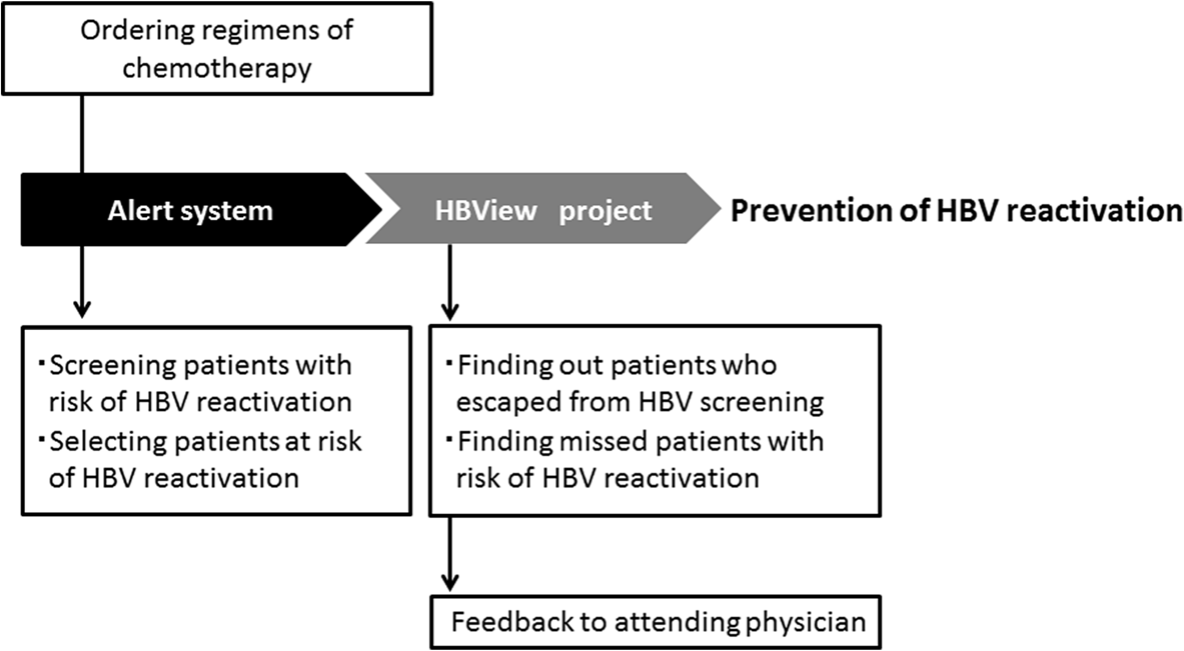
Diagram summarizing the alert system and the HBView project. HBV, hepatitis B virus

The HBView project was initiated in January 2014 at our hospital pharmacy to prevent HBV reactivation. The schema of the measures taken to prevent HBV reactivation is shown in Fig. 2. The purpose of the project was to investigate why patients eluded HBV screening and why that fact sometimes went unnoticed.

We reviewed hepatitis B surface antigen (HBsAg), HBsAb, HBcAb, and HBV-DNA test results as well as the corresponding patients’ histories and the number of regimen cycles that they underwent. We inquired with the attending physician if the HBV screening test was not ordered according to the JSH Guidelines for the Management of Hepatitis B Virus Infection [5] at the time the chemotherapy regimen was ordered.

### Study design and ethics statement

This study was retrospective, and data regarding the basic characteristics, cancer type, chemotherapy regimen, and status of HBV infection for all patients undergoing chemotherapy were collected; physician and pharmacist charts were also reviewed. The study was approved by the Ethics Committee and the Institutional Review Board of the Nagoya City University Hospital (No. 1004).

Data on HBsAg, HBsAb, HBcAb, and HBV-DNA in patients who had been prescribed new regimens prior to the introduction of the alert system (September 2010 to March 2012) and afterwards (April 2012 to October 2013) were collected. The inspection rates of HBsAb and/or HBcAb and HBV-DNA according to the JSH Guidelines for the Management of Hepatitis B Virus Infection [5] were determined.

We also performed a retrospective investigation of pharmacists’ interventions in patients who were prescribed regimens prior to the start of the HBView project (July 2013 to December 2013) and after it commenced (January 2014 to June 2014) using electronic medical records. In addition, data on HBsAg, HBsAb, HBcAb, and HBV-DNA in patients who had been prescribed new regimens prior to start of the HBView project (July 2013 to December 2013) and afterwards (January 2014 to June 2014) were reviewed.

### Statistical analysis

Results are expressed as the mean ± standard deviation or as numbers and percentages. Categorical variables were compared by Fisher’s exact test or the chi-squared test, and the means of two groups were compared using the unpaired *t*-test. Significance was set at *P* < 0.05. Statistical analysis was performed with R (The R Foundation for Statistical Computing, version 3.1.1) [14].

## Results

### Patient characteristics

The number of patients who were prescribed new regimens was 880 and 926 before and after the alert system was established, respectively. There was no significant difference in patient characteristics between the two groups (Table 1).

**Table 1:** Patient characteristics in the groups studied before and after alert system implementation

	Pre-alert system	Post-alert system	*P*-value	Analysis
	(*n* = 880)	(*n* = 926)		
Age (years; mean ± SD)	62.0 ± 13.8	62.3 ± 15.3	0.137	(1)
Sex (male/female)	465/415	488/438	0.952	(2)
Cancer type (number, %)				
Breast cancer	119 (13.5)	124 (13.4)	0.99	(2)
Lung cancer	129 (14.7)	141 (15.2)	0.785	(2)
Gastric cancer	43 (4.9)	36 (3.9)	0.356	(2)
Colorectal cancer	59 (6.7)	61 (6.6)	0.996	(2)
Hematopoietic malignancy	151 (17.2)	184 (19.9)	0.155	(2)
Others	379 (43.1)	380 (41.0)	0.409	(2)

(1) Unpaired t-test, (2) Chi-squared test. SD, standard deviation

### Compliance with HBV screening guidelines with the alert system

**Fig. 3:**
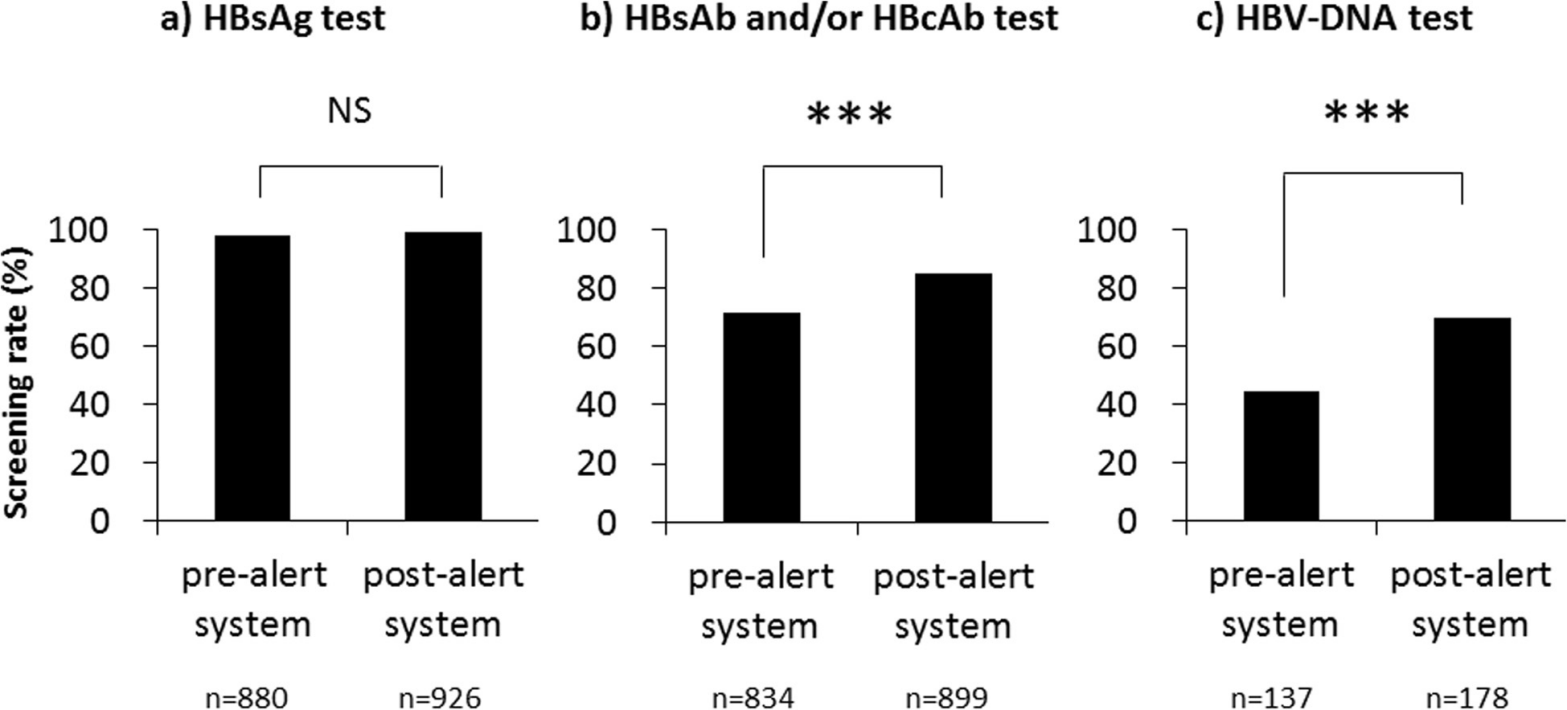
Screening rates of hepatitis B virus (HBV) before and after introducing the alert system. **a**) represents HBsAg test. **b**) represents HBsAb and/or HBcAb test. **c**) represents HBV-DNA test. HBsAb, hepatitis B surface antibody; HBsAg, hepatitis B surface antigen; HBcAb, hepatitis B core antibody, NS: not significant, ***P  <  0.001 (Chi-squared test)

As shown in Fig. 3, the screening rates for HBsAg, HBsAb and/or HBcAb, and HBV-DNA were 98.0 % (862/880), 71.6 % (621/834), and 44.5 % (61/137), respectively, before the introduction of the alert system. After introducing the alert system, the screening rates for HBsAg, HBsAb and/or HBcAb, and HBV-DNA were 99.0 % (917/926), 84.9 % (777/899), and 69.7 % (124/178) respectively. There was no significant difference in screening rates for HBsAg before and after the alert system was initiated (*P* = 0.060) (Fig. 3a). However, the screening rates for HBsAb, HBcAb, and HBV-DNA after the introduction of the alert system increased compared to the rates prior to its introduction (*P* < 0.001) (Fig. 3b, c).

### Pharmacist intervention concerning HBV reactivation

**Fig. 4:**
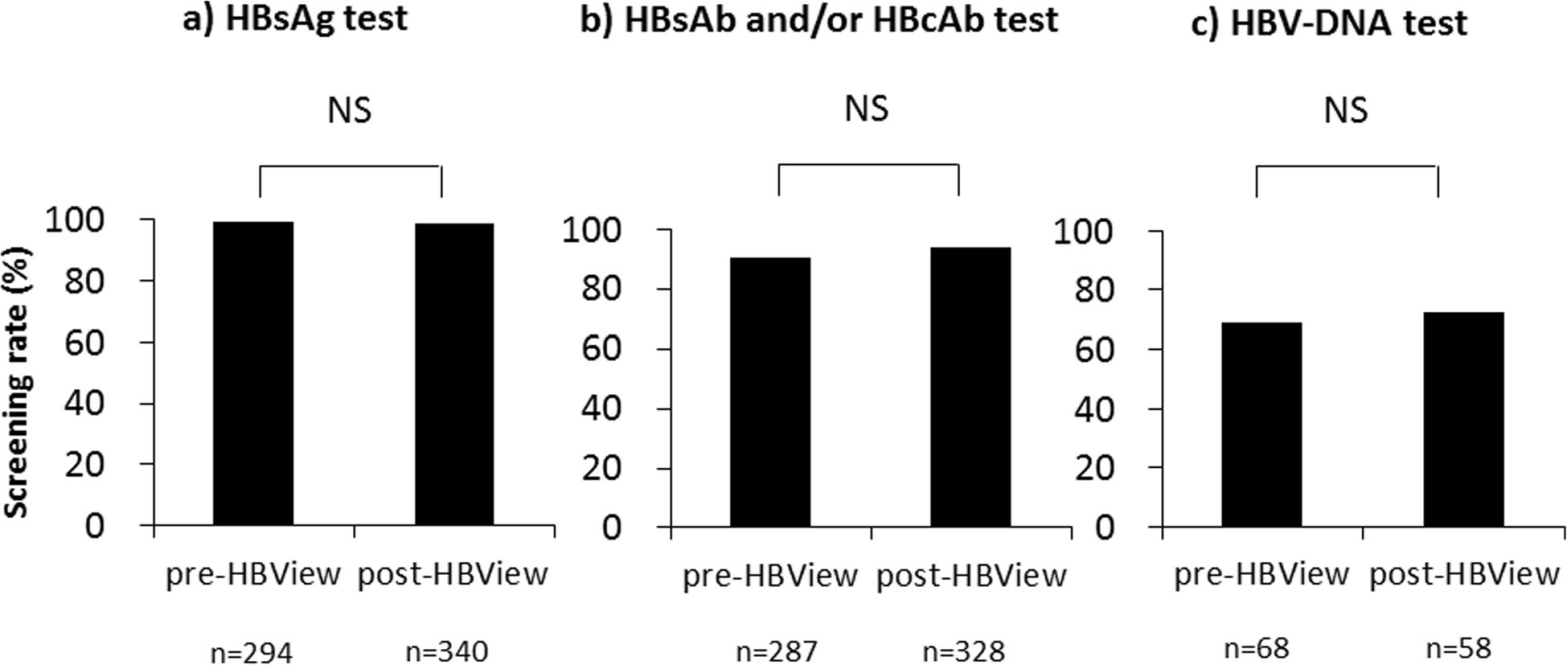
Screening rates of hepatitis B virus (HBV) before and after the start of the HBView project. **a**) represent HBsAg test. **b**) represents HBsAb and/or HBcAb test. **c**) represents HBV-DNA test. HBsAb, hepatitis B surface antibody; HBsAg, hepatitis B surface antigen; HBcAb, hepatitis B core antibody, NS: not significant (Chi-squared test)

The number of patients who were prescribed new regimens was 294 and 340 before and after the HBView project was established, respectively. There was no significant difference in patient characteristics between the two groups (data not shown). As shown in Fig. 4, the screening rates for HBsAg, HBsAb and/or HBcAb, and HBV-DNA were 99.0 % (291/294), 90.6 % (260/287), and 69.1 % (47/68) respectively, before the start of the HBView project. After the start of the HBView project, the screening rates for HBsAg, HBsAb and/or HBcAb, and HBV-DNA were 98.2 % (334/340), 93.6 % (307/329), and 72.4 % (42/58), respectively. The number of regimens reviewed by pharmacists was 5208 and 5228 before and after the initiation of the HBView project, respectively. Pharmacists intervened six times in patient cases before the HBView project was initiated; however, the number of interventions increased to 17 after the project commenced (*P* = 0.022).

Pharmacists had not exposed any incidents of serious oversight prior to the HBView project. However, two cases were exposed after the project was initiated (Table 2). One was a 58-year-old woman with cervical cancer who was to undergo surgery concomitant with radiotherapy and CDDP (cisplatin 40 mg/m^2^/2 h) 4 days later. In reviewing the regimen, a pharmacist noticed that her HBsAg and HBV-DNA levels were unknown even though her HBsAb and HBcAb tests were positive. The pharmacist informed the attending physician, and an HBV-DNA test was ordered. The test revealed a high copy number of HBV-DNA (7.4 log copies/mL). The attending physician immediately consulted a hepatologist, and nucleic acid analog treatment was started. Levels of alanine transaminase and aspartate transaminase increased temporarily, but there was no HBV reactivation. HBV-DNA levels gradually subsided.

**Table 2:** The number of interventions concerning HBV reactivation by pharmacists

	Pre-HBView	Post-HBView	*P*-value	Analysis
	(n = 5208)	(n = 5228)		
The number of interventions concerning HBV reactivation	6	17	0.022	(1)
	Pre-HBView	Post-HBView	*P*-value	Analysis
	(*n* = 6)	(*n* = 17)		
The number of initially unnoticed patients discovered to be at risk for HBV reactivation	0	2	0.538	(2)

(1) Chi-squared test, (2) Fisher’s exact test. HBV, hepatitis B virus

The other patient was a 72-year-old man with colorectal cancer who had undergone partial hepatectomy 4 years previously. HBsAg was positive before surgery; however, he had been receiving mFOLFOX6 (oxaliplatin 85 mg/m^2^/2 h, levofolinate 200 mg/m^2^/2 h, and fluorouracil 400 mg/m^2^ bolus + 2400 mg/m^2^/46 h), bevacizumab (5 mg/kg) + mFOLFOX6, and bevacizumab + FOLFIRI (irinotecan 150 mg/m^2^/1.5 h, levofolinate 200 mg/m^2^/2 h, and fluorouracil 400 mg/m^2^ bolus + 2400 mg/m^2^/46 h) without preventive treatment with a nucleic acid analog. Fortunately, the patient had not experienced HBV reactivation. After commencement of the HBView project, a pharmacist noticed that this patient had tested positive for HBV-DNA 1 year previously. The pharmacist suggested that the attending physician consult a hepatologist. HBV-DNA positivity was confirmed; yet, the attending physician took no action. Two months later, another pharmacist noticed the patient’s HBV-DNA positive status and informed the attending physician again. A nucleic acid analog was administered to the patient, after which HBV-DNA gradually decreased.

## Discussion

In recent years, HBV reactivation in cancer chemotherapy has attracted more attention, and there have been reports of certain measures being implemented to address this problem [11–13, 15]. For example, a system was designed to encode the status of patients receiving immunosuppressive therapy and/or chemotherapy, in which the system automatically checks to determine if the patient has undergone the required tests and treatments according to the practical guidelines for hepatitis B and reports the results to the primary physician [15]. Additionally, there have been other anti-HBV reactivation measures that utilize alert systems in Japan and other countries [11–13]. In this investigation, we evaluated the alert system at our hospital, and found that it greatly improved the inspection rates of HBsAb and/or HBcAb, as well as of HBV-DNA, according to the JSH Guidelines for the Management of Hepatitis B Virus Infection [5]. The alert system was very effective in helping physicians in multiple clinical departments to recognize the need for such inspections, although enforcement was not perfect. While the automated alert system is very useful for risk management, it may be insufficient for ensuring screening compliance. Therefore, we initiated the HBView project. The HBView project did not significantly improve compliance with HBV screening (Fig. 4). There are some possible causes for this. The initial alert system already had greatly improved compliance with HBV screening, which may have contributed most to the HBView project’s non-significant results. We determined that intervention with further compelling force was necessary to improve the compliance rate for HBV screening.

While there were no significant differences in patient characteristics before and after the HBView project was established, pharmacists discovered two patients at risk of HBV reactivation after the start of HBView project who were not noticed beforehand (Table 2). In the first case, a pharmacist discovered that the HBV screening result was missing. HBV reactivation may very well have been prevented by the pharmacist’s quick action to rectify the situation. In the second case, the patient was found to have been HBsAg-positive at preoperative inspection after having started chemotherapy. Awareness and recognition of HBV reactivation by the medical staff at our hospital may have been low in days prior to the introduction of the alert system. However, this case was overlooked after the alert system was introduced, probably because the system was not yet functioning effectively. The intervention of the pharmacists after the HBView project was initiated may very well have prevented HBV reactivation.

It is already recognized that HBV screening during cancer chemotherapy is low [8–10]. However, this study suggests that oversight can easily occur even if HBV screening is performed. Furthermore, the study revealed that there is a large risk associated with the failure to notice and/or report positive HBV results. It was reported that the proportion of HBsAg-positive patients was 1.5 % and that of HBsAg-negative patients with HBcAb and/or HBsAb-positivity was 23.2 % during inspection for HBV before blood transfusions [16]. It was also reported that evidence of resolved hepatitis B existed in 31.5 % of rheumatoid arthritis patients at other facilities [17]. Therefore, it is suspected that HBV-infected patients exist across many medical disciplines. The risk of HBV reactivation underlies the cancer treatment field and is of widespread concern, and must therefore be adequately addressed. An interesting report on HBV screening was published recently [18]. In that report, a high compliance rate of adequate screening and prophylactic therapy was achieved by introducing a compulsive computerized order entry-based alert system. However, the strength of such intervention by an alert system is likely to be controversial in different institutions or regions.

Detailed information about the background and risk factors of HBV reactivation is unknown. The JSH Guidelines for the Management of Hepatitis B Virus Infection are as follows: “When immunosuppressive therapy or chemotherapy is administered to HBsAg-positive inactive carriers, or to patients with resolved HBV infection and HBV DNA levels of 2.1 log copies/mL on pretreatment screening tests, nucleic acid analog therapy should be commenced without delay” [5]. HBV reactivation is known to progress to fulminant hepatitis. Measuring HBV DNA levels in the serum is useful for determining the status of chronic HBV infection, as it differentiates between active and inactive disease states. On average, there are approximately 19 weeks between the increase in HBV-DNA levels and the onset of hepatitis [1]. This duration is the reason for the time interval between the first and second message in the alert system. The risk factors for HBV reactivation include the nature of treatment, such as hematopoietic stem cell transplantation, organ transplantation, and combination therapy of rituximab and corticosteroid or systemic chemotherapy; and the status of HBsAg, HBsAb, and/or HBcAb positivity [16]. Other risk factors continue to be investigated [1, 2, 19–21]. Further investigations will clarify additional therapeutic and prognostic aspects of HBV reactivation.

Medication-use evaluation (MUE) has long been regarded as important for improving medication-use processes with the goal of optimal patient outcomes [22]. The present study suggests that the MUE processes conducted by pharmacists prevented medication-related problems and improved patient safety. It is recommended that pharmacists play a wider role in risk management during medical therapy. In the field of cancer treatment, pharmacists manage medications, accept consultations, and confirm that regimens are correct on a daily basis. The HBView project highlighted the importance of pharmacists with respect to HBV reactivation prevention and is the type of endeavor that can both clarify HBV reactivation consequences and improve outcomes going forward.

## Conclusions

Detection of patients at risk for HBV reactivation was insufficient when using only an automatic alert system. An additional system of verification and intervention by pharmacists provided greater assurance of detecting such patients.

## Abbreviations

HBV: Hepatitis B virus
JSH: The Japan society of hepatology
HBsAg: Hepatitis B surface antigen
HBsAb: Hepatitis B surface antibody
HBcAb: Hepatitis B core antibody
MUE: Medication use evaluation
